# Psychological impact and associated factors of the COVID-19 pandemic among pregnant women in Fafan Zone health institutions, Somali Region, Eastern Ethiopia, 2021

**DOI:** 10.1186/s12905-024-03109-9

**Published:** 2024-04-30

**Authors:** Meka Kedir Jibril, Ahmed Adem Yimam, Neima Ridwan Abdu, Seid Yimam Ali

**Affiliations:** 1 School of Nursing and Midwifery Department of Nursing, Integrated Clinical and Community Mental Health Jigjiga University College of Medicine and Health Science, Jigjiga, Ethiopia; 2 Department of Internal Medicine, Jigjiga University Comprehensive Specialized Hospital and College of Medicine and Health Science School of Medicine, Jigjiga, Ethiopia; 3 Warabe University College of Medicine and Health Science Department of Midwifery, Warabe, Ethiopia; 4 Jigjiga University College of Medicine and Health Science School Medicine, Jigjiga, Ethiopia

**Keywords:** Coronavirus, Outbreak, Psychological Impact, Pregnant women, Associated factors

## Abstract

**Background:**

Despite pregnant women's vulnerability to respiratory illnesses and pregnancy complications during the COVID-19 pandemic, research on its psychological impact in the study area, is limited.

**Objective:**

This study aims to fill this gap by examining the prevalence and factors linked to the psychological impact among pregnant women in the Fafan zone, Somali region of Ethiopia.

**Methods:**

A cross-sectional study conducted from April 1^st^ to April 30^th^, 2021, randomly selected health facilities for inclusion. The Impact of Event Scale-Revised (IES-R) assessed psychological impact, and data were analyzed using SPSS V 22. Variables with a *p*-value ≤ 0.25 in bivariate analysis were considered for multivariate analysis via multiple logistic regressions with the backward elimination method.

**Results:**

The study involved 294 pregnant women, constituting 73% of the respondents. The prevalence of psychological impact attributed to the COVID-19 pandemic was 27.2%. Factors such as being in the first trimester of pregnancy (AOR: 5.32), travel history to infected areas (AOR: 3.71), obtaining COVID-19 information from television (AOR: 4.81), and using social media for 1 to 2 hours daily for updates (AOR: 1.35) were significantly associated with this impact.

**Conclusion:**

While the psychological impact among pregnant women in this study was relatively lower compared to other research, factors such as gestational age, TV media exposure, travel history, and social media usage for COVID-19 updates were strongly linked to this impact, highlighting the necessity for psychological support services for pregnant women during challenging times.

## Introduction

On January 12, 2020, the World Health Organization (WHO) declared the coronavirus disease 2019 (COVID-19), which originated in Wuhan in December 2019, as a pandemic [1]. Fast forward to December 2021, the global burden has exceeded 266 million cases and 5 million deaths globally, with Africa bearing a significant burden, evidenced by a case fatality rate (CFR) of 2.36%. Ethiopia alone has recorded nearly 400,000 cases and thousands of lives lost [2].

The COVID-19 pandemic has not only impacted physical health but also mental well-being worldwide. Fear of the virus, prolonged quarantines, economic crises, and the halt of public transportation have heightened stress and anxiety levels, increasing the risk of mental health disorders globally [3–5].

One particularly vulnerable group could be pregnant women due to their condition [6, 7]. Relevant studies indicated that pregnant women are at higher risk of contracting COVID-19 due to their susceptibility to respiratory illnesses such as; SARS and MERS and potential impact on pregnancy outcomes [8–10]. Subsequently, attention to their psychological well-being during the pandemic is crucial.

Pregnancy brings about physical and psychological changes that directly affect their mental health. On top of that Pandemic-related uncertainties have intensified the burden on pregnant mothers [11, 12]. Limited access to regular check-ups adds to their stress and anxiety, compounded by concerns about their baby’s health and reported increases in premature births, stillbirths, and miscarriages since the pandemic began [9, 13–17] . Moreover, COVID-19 can affect both the mother's and the baby's health by changing how their immune systems work [9].

Emerging evidence indicates increased levels of anxiety, depression, and stress among pregnant women during the pandemic [11, 18, 19]. Studies show an increased prevalence of anxiety and depression among them, with females being more prone to developing symptoms such as depression, anxiety, insomnia, and stress [11, 18–21].

The global impact of the COVID-19 pandemic poses significant health risks, particularly for pregnant women [15]. Despite attention to concerns like vertical transmission and fetal infection, there is a clear gap in addressing the mental health aspects of pregnancy. Research specifically on the psychological impact of the pandemic on pregnant women is limited, especially in Ethiopia. Therefore, the main problem addressed by this study is the lack of comprehensive understanding regarding the psychological effects of the COVID-19 pandemic on pregnant women in Ethiopia, particularly in the study area.

This study aims to address this gap by investigating the prevalence and associated factors of psychological impact among pregnant women receiving antenatal care at Fafan Zone health institutions. By doing so, we aim to fill this critical knowledge gap and contribute to the development of targeted interventions to support the mental well-being of pregnant women.

## Materials and methods

### Study area

Fafen Zone is situated in the Somali Regional State, approximately 628 kilometers away from Ethiopia's capital city, Addis Ababa. It comprises 16 health centers and 2 hospitals. According to the 2014 Census conducted by the Central Statistical Agency of Ethiopia (CSA), the total population of this zone is 1,190,794, with 616,810 men and 541,794 women. Among them, 21.6% of the population, or 257,556 people, reside in urban areas, while 78.4% of the population, or 933,240 individuals reside in rural [22].

### Study design and period

A cross-sectional survey was conducted in eight selected health institutions within the Fafen Zone from April 1^st^ to April 30^th^, 2021. The survey focused on pregnant women attending antenatal care (ANC) follow-up visits.

### Eligibility criteria

The study enrolled apparently healthy pregnant women of any gestational age who visited the selected health institutions during the study period. However, pregnant women who were critically ill, in active labor, or unable to communicate verbally or audibly during data collection were excluded

### Sample size determination and sampling technique

The sample size for this study was determined using the single population proportion method due to the lack of previous research in Ethiopia with a similar setting. Assuming an expected proportion of psychological impact due to COVID-19 in the Fafen Zone to be 50% (*p*=0.5), a margin of error (d) of 5%, and a standard normal deviation at 95% confidence interval (Z) of 1.96, with a non-response rate of 5%, the final sample size was calculated to be 403.

From the 16 health institutions in the study area, 8 were randomly selected using a simple random sampling technique. The sample size was then proportionally allocated to each selected health institution based on their average number of ANC service users over the previous three consecutive months, which was 3050 in total. This corresponded to an average of 1017 ANC service users per month. Participants were randomly selected from each health institution using a simple random sampling method.

### Data collection instruments

Data collection involved structured and semi-structured questionnaires covering sociodemographic details, psychological impact, social support, pregnancy experiences, and healthcare factors. Psychological impact was assessed using the Impact of Events Scale-Revised (IES-R) was employed. This self-administered questionnaire consisted of 22 items organized into three clusters of symptoms. The IES-R is a suitable instrument for assessing subjective responses to specific traumatic events among older adults, focusing on intrusion, avoidance, and hyperarousal. Eight items measured intrusions, eight items measured avoidance, and six items measured hyperarousal. Each item was scored from 0 to 4, indicating the degree of impact experienced by participants. Scores ranged from 0 to 88, with higher scores indicating greater psychological impact. Based on the total score, pregnant women were categorized as follows: 0-23 (no psychological impact), 24-32 (mild), 33-36 (moderate), and greater than 36 (severe psychological impact) [23]. The IES-R demonstrated strong internal consistency (Cronbach's α: 0.79 to 0.91) and has been validated in Ethiopia (Cronbach’s α > 0.9) [24, 25].

Social support was assessed using the Oslo-3 Social Support Scale (OSS-3), which consisted of three questions. Response categories were evaluated independently for each question, and a sum score was calculated by aggregating the raw scores. The Oslo-3 Social Support Scale (OSS-3) tool has been used in previous community and facility-based studies in Ethiopia and demonstrated good utility [26, 27]. In this study, OSS-3 scores were analyzed both as a sum score and on an item-by-item basis, with three overarching categories: "poor support" (3–8), "moderate support" (9–11), and "strong support" (12–14) [28].

### Data quality assurance

The questionnaire was translated from English to the local Somali language by a language expert and then back-translated into English to ensure accuracy and consistency. The translated Somali questionnaire was used for data collection. Prior to the study, a comprehensive two-day training session was conducted for the data collectors (ten BSc midwives) and supervisors (four BSc Public Health trained professionals). The training focused on the study objectives, basic interview techniques, and ethical considerations. The principal investigators supervised the training to ensure its effectiveness. Data collectors also received guidance from the supervisors and principal investigators to ensure the completeness and clarity of the questionnaire. Furthermore, a pretest was conducted on 19 (5% of the sample) clients at Dagahabur Hospital.

### Data processing and analysis

First, the collected data underwent checks for completeness and consistency. Subsequently, it was coded and entered into Epi Info V3.5.3. The data was then exported and analyzed using SPSS V 22. Descriptive statistics were used to determine the prevalence of psychological impact during the COVID-19 pandemic among pregnant women. Binary logistic regressions were conducted to assess the relationship between predictors and psychological impact. Significant variables (*P* < 0.25) from the binary logistic regression models were selected, and a multivariable logistic regression model was developed to identify independent predictors of psychological impact during the COVID-19 pandemic. The strength of the association was measured using odds ratios with 95% confidence intervals, and statistical significance was considered at *P* < 0.05.

### Ethical consideration

Prior to the study, ethical clearance was obtained from the office of research and community service of Jigjiga University and formal support letters from relevant administrations. Informed consent was obtained from all pregnant participants, involving transparent disclosure of the study's purpose, potential risks, and benefits, thereby securing their voluntary participation. Critical measures were implemented to ensure confidentiality, including protection of personal data and restricted access to authorized personnel only were critically implemented. Data obtained during the study was kept confidential. The researchers had taken steps to minimize any potential risks to the participants of pregnant women, such as psychological impact or discomfort, and provided appropriate support.

## Results

### Sociodemographic characteristics

A total of 294 respondents participated in this study, resulting in a response rate of 73%. Among the participants, 289 individuals (98.3%) were married and living together with their husbands. Regarding education level, 179 participants (60.9%) were classified as illiterate. Additionally, more than ninety percent of the study subjects were residents of urban areas (Table 1).

**Table 1:** Distribution of sociodemographic factors among pregnant women attending antenatal care at Fafan Zone Health Institutions, Somali Region Eastern Ethiopia in 2021 (*n*= 294)

**Variables**	**Category**	**Frequency**	
		**Number**	**Percent (%)**
Age	<25 Years	116	39.53
	>25 Years	178	60.47
Marital status	Married	289	98.30
	Unmarried	2	0.70
	Divorced	3	1.00
Residence	Urban	272	92.5
	Rural	22	7.5
Religion	Muslim	251	85.4
	Orthodox	36	12.2
	Protestant	7	2.4
Education	Illiterate	179	60.9
	Informal Education	13	4.4
	Primary Education	30	10.2
	Secondary	34	11.6
	Diploma and Above	38	12.9
Occupation	House wife	240	81.6
	Pastoralist	10	3.4
	Merchant	7	2.4
	Government Employee	26	8.8
	Private	9	3.1
	Daily Labor	2	0.7
Income	< 2500 Birr	270	91.8
	>2500 Birr	24	8.2

### Pregnancy and clinical-related factors

The majority of the study participants (86.7%) were classified as multigravida, indicating that they had been pregnant more than once before. Additionally, 273 participants (92.9%) had a gestational age greater than 16 weeks at the time of the study. All study participants reported no history of postpartum psychiatric disorders or antenatal depression (Table 2).

**Table 2:** Distribution of pregnancy and clinical-related factors among pregnant women attending antenatal care at Fafan Zone Health Institutions, Somali Region Eastern Ethiopia in 2021 (*n* = 294)

**Variables**	**Categories**	**Frequency**	
		**Number**	**Percent (%)**
Gravidity	Primigravida	39	13.3
	Multigravida	255	86.7
Gestational age	<16wks	21	7.1
	>16wks	273	92.9
Headache	Yes	112	38.1
	No	182	61.9
Sore throat	Yes	15	5.1
	No	279	94.9
Body ache	Yes	16	5.4
	No	278	94.6
Fever	Yes	23	7.8
	No	271	92.2
Breathing difficulty	Yes	23	7.8
	No	271	92.2
Hypertension	Yes	27	9.2
	No	267	90.8
Asthma	Yes	22	7.5
	No	272	92.5

### Non-clinical related factors

**Fig. 1:**
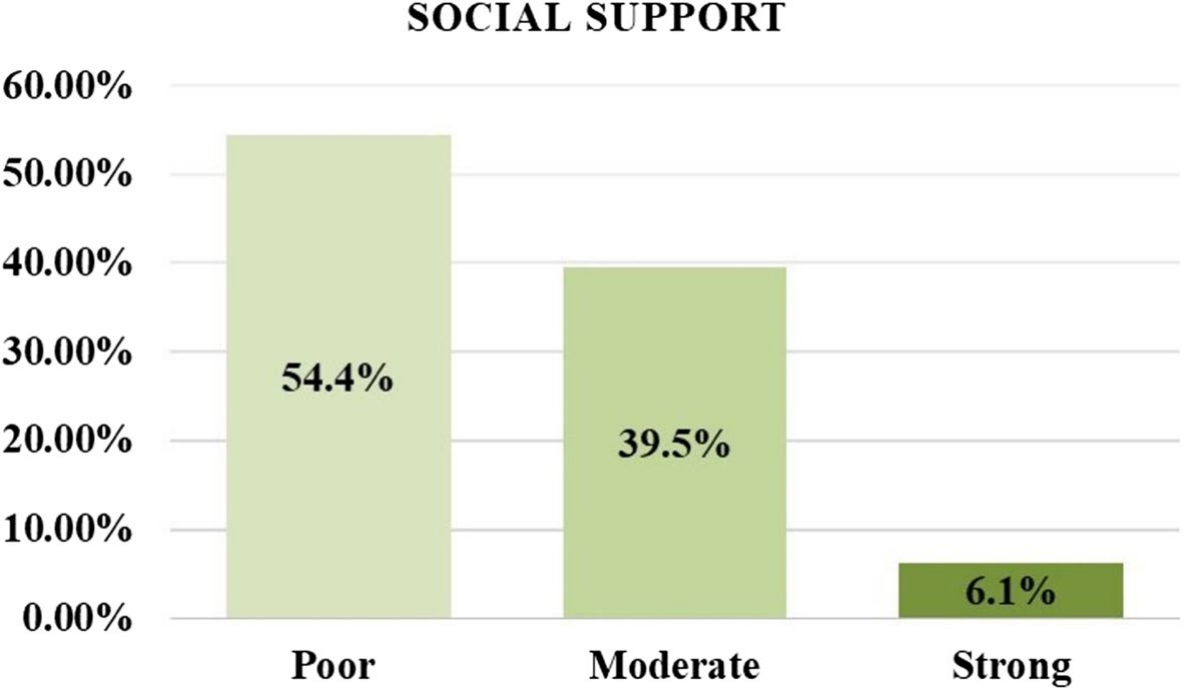
Subtypes of social support among pregnant women attending antenatal care at Fafan Zone Health Institutions, Somali Region Eastern Ethiopia in 2021 (*n*= 294)

The findings of this study reveal that among the study participants, 195 individuals (66.3%) experienced an average waiting time of more than 30 minutes at the health facility to receive ANC services. Additionally, 23 participants (7.8%) reported facing problems related to COVID-19 during their ANC visits (Table 3) & (Fig. 1).

**Table 3:** Distribution of non-clinical related factors among pregnant women attending antenatal care at Fafan Zone Health Institutions, Somali Region Eastern Ethiopia in 2021 (*n* = 294)

**Variables**	**Categories**	**Frequency**	
		**Number**	**Percent (%)**
Average waiting time in the health facility to receive ANC service	< 30 minutes	99	33.7
	> 30 minutes	195	66.3
Contact history/ travel to infected area	Yes	23	7.8
	No	271	92.2
Social media usage	Not using social media	143	48.6
	Less than 2 hrs. per day	102	34.7
	More than 4 hrs. per day	49	16.7
Watching television to get COVID‐19 information	Less than 2 hrs. per day	160	54.4
	2‐4 hrs. per day	111	37.8
	More than 4 hrs. per day	23	7.8

### Prevalence of psychological impact of COVID-19 pandemic among pregnant women

**Fig. 2:**
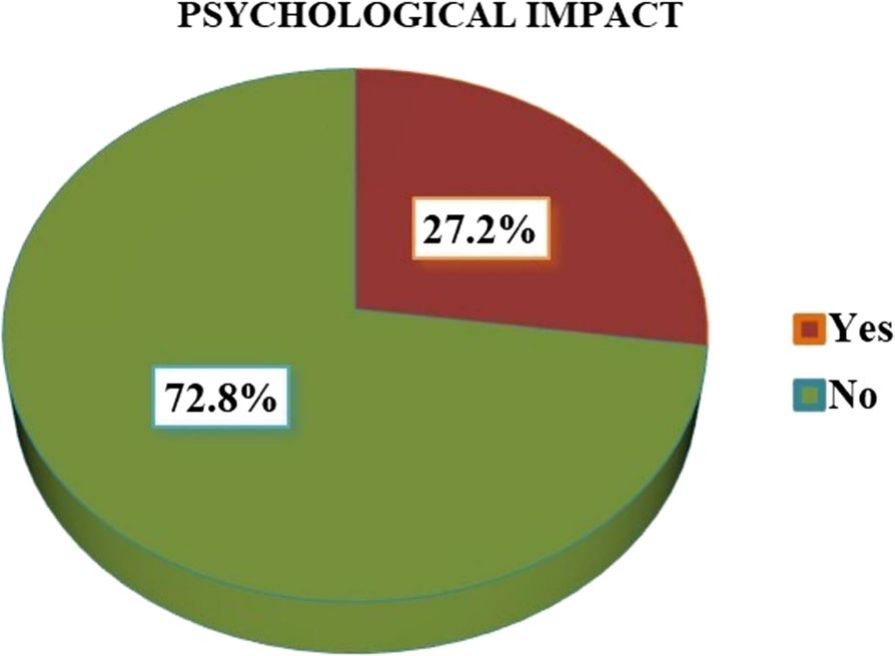
Prevalence of psychological impact of COVID-19 pandemic among pregnant women attending antenatal care at Fafan Zone Health Institutions, Somali Region Eastern Ethiopia in 2021 (*n*= 294)

In this study, we found that the prevalence of psychological impact due to the COVID-19 pandemic among pregnant women was determined to be 27.2% (Fig. 2).

### Factors associated with psychological impact of COVID-19

Logistic regression analysis was conducted to identify factors associated with the psychological impact of COVID-19 among pregnant women. In the bivariate logistic regression, variables including age, gravidity, gestational age, TV viewing duration, travel to infected areas, COVID-19 related health education, and obtaining information through social media showed associations with psychological impact (*p*-value ≤ 0.25). However, factors like marital status, residence, religion, education, occupation, income, chronic medical conditions, and mental illness did not exhibit an association.

Further analysis using multivariate logistic regression revealed significant associations. Pregnant women with a gestational age of 16 weeks or less were five times more likely to experience psychological impact due to COVID-19 compared to those with a gestational age greater than 16 weeks (AOR = 5.32, 95% CI: 1.79, 15.82). Additionally, women who watched television for two to four hours per day during the COVID-19 outbreak were nearly five times more likely to experience psychological impact compared to those who watched television for less than two hours per day (AOR = 4.81, 95% CI: 1.50–15.38). Similarly, mothers who had a history of contact with infected areas through travel were four times more likely to experience psychological impact compared to women who had no history of travel to infected areas (AOR = 3.71, 95% CI: 1.17–11.69). Lastly, although pregnant women who used social media to obtain information about COVID-19 for one to two hours per day were twice as likely to experience psychological impact compared to mothers who did not use social media for COVID-19 information (AOR: 1.35 (0.51, 3.53) in bivariate model), this association did not persist in multivariate analysis (Table 4).

**Table 4:** Bivariate and multivariate logistic regression of statistically significant variables in Bivariate model among pregnant women attending antenatal care at Fafan Zone Health Institutions, Somali Region Eastern Ethiopia in 2021 (*n* = 294)

**Variables**	**Category**	**Psychological impact**		**COR (95% CI)**	**AOR (95% CI)**	***P*- value**
		**Yes**	**No**			
**Age (years)**	≤25	25 (21.6%)	91 (78.4%)	1.63 (0.94,2.80)	0.95 (0.48, 1.86)	
	>25	55 (30.9%)	123 (69.1%)	1	1	
**Gravidity**	Primi	6 (15.4%)	33 (84.6%)		0.73 (0.25, 2.14)	
	Multi	74 (29.0%)	181 (71.0%)	1	1	
**Gestational in (wks.)**	≤16	11 (52.4%)	10 (47.6%)	3.25 (1.32, 7.99)	5.32 (1.79, 15.82)	0.003
	>16	69 (25.3%	240 (74.7%)	1	1	
**Watching TV (hrs.)**	<2	26 (16.2%)	134 (83.8%)	1	1	0.008
	2-4	43 (38.7%)	68 (61.3%)	724 (1.88, 11.85)	4.81 (1.50, 15.38)	
	>4	11 (47.8%)	12 (52.2%)	1.45 (0.58, 3.57)	2.31 (0.77, 6.94)	
**Travel to infected area**	Yes	10 (47.6%)	11 (52.4%)	2.64 (1.07, 6.47)	3.701 (1.17, 11.69)	0.025
	No	70 (25.6%)	203 (74.4%)	1	1	
**COVID-19 health education**	Yes	33 (22.3%)	115 (77.7%)	1	1	
	No	47 (32.2%)	99 (67.8%)	1.65(0.98, 2.78)	1.45 (0.78, 2.68)	
**Social media to get information (hrs.)**	No	26 (18.2%)	117 (81.8%)	1	1	0.045
	≤2	37 (36.3%)	65 (63.7%)	2.39 (1.15, 4.93)	1.35 (0.51, 3.53)	
	>2	17 (34.7%)	32 (65.3%)	0.93 (0.45, 1.90)	0.55 (0.23, 1.33)	

*AOR* Adjusted Odds Ratio, *COR* Crude Odds Ratio, *CI* Confidence Interval

## Discussion

The objective of this study was to investigate the mental health status of pregnant women among the COVID-19 pandemic. While pregnancy is often accompanied by psychological impact [11, 18, 19], the emergence of COVID-19 has amplified concerns about its potential effects on both maternal health and childbirth outcomes [9, 13–17].

The overall prevalence of psychological impact in this study was 27.2%. Comparable rates were observed in studies conducted in Italy 28.6% [29], Eastern China (27.9%) [30], Northern China (30.6%) [18], and Saudi Arabia (23.6%) [31]. However, the prevalence of psychological impact in this study is relatively lower compared to studies conducted in Colorado (60%) [32], Canada (37%) [13], Greece 48.3% [21], Turkey (52.7%) [33], China (53.8%) [34], and Mettu (Ethiopia) (45.1%) [24]. On the other hand, the prevalence of psychological impact in this study is higher than that reported in Belgium 13.6 [35], Iran (19.3%) [36] and Addis Ababa (Ethiopia) 21.5% [37]. The observed variations in the prevalence of psychological impact across different studies can be attributed to several factors, including differences in sample size, sociodemographic characteristics, and cultural backgrounds and the survey tools used [30].

Many factors were found to be potentially related to psychological impact including, gestational age, television viewing habits, travel to infected areas, and obtaining COVID-19 information through social media. Research suggests that psychological impact due to COVID-19 is increased among women in the first trimester of pregnancy. Consistent with these findings, the current study revealed that women in their first trimester experienced the highest levels of psychological impact. The odds of experiencing psychological impact were five times higher in pregnant women in their first trimester. This aligns with studies conducted in Italy and Northern Iran, indicating that the early stages of pregnancy may be particularly vulnerable to psychological impact during the COVID-19 pandemic [38]. Additionally, women with a gestational age of less than 20 weeks had a higher risk of psychological impact, as observed in study conducted in Northern Iran. In these study, gestational age of less than 20 weeks remained significant factors in the multivariate analysis, emphasizing their importance in understanding and addressing psychological impact among pregnant women during the pandemic [36].

A significant association was discovered between contact history, particularly travel to infected areas, and psychological impact. Pregnant women with a history of travel to infected areas were about four times more likely to experience psychological impact. This finding contrasts with a study conducted in India, which found no statistically significant association between contact history and psychological health impact [39].

This study revealed that watching television for 2-4 hours daily was significantly linked to psychological impact among pregnant women. Those pregnant mothers who watch TV for 2-4 hours were five times more likely to experience impact compared to non-viewers. Similar to findings from Japan, excessive media exposure during health crises like 9/11, Ebola, and natural disasters consistently leads to increased fear and poorer mental health. This trend is also observed with COVID-19, emphasizing the importance of limiting media intake and relying on reliable sources for better mental well-being [40]. However, studies from Turkey indicate that specific, accurate health information may reduce psychological impact during outbreaks. This variation may be attributed to differences in program content and cultural perceptions of television's influence on mental well-being [33].

In our study, we found that obtaining COVID-19 information through social media was associated with a 1.35 times increase in psychological impact. This aligns with previous research in USA shown that young adults experience lower depression symptoms when they receive more offline emotional support and less online informational support [41]. Similarly, in South Korea, excessive time spent on social media platforms was linked to a higher likelihood of experiencing anxiety and depression symptoms [42]. Indian research also supports these findings, indicating that increased exposure to COVID-19 information through mass and social media is strongly associated with significant psychological health issues. Addressing the harmful effects of social media exposure during the pandemic is an urgent international public health priority to safeguard the psychological well-being of vulnerable groups [43].

## Limitations of the study

While this study was conducted across multiple settings and participants were recruited using a probability sampling technique, generalizing the findings may be challenging due to the cross-sectional nature of the study. Additionally, the psychometric properties of the tools used were validated in Ethiopia, enhancing the reliability of the study's measurements.

It's important to note that the study did not assess the detailed income levels of participants, which could potentially impact their living situations. This missing information could have implications for understanding the socioeconomic factors influencing psychological impact among the study subjects.

## Conclusion

The study revealed that one-third of pregnant women experienced moderate to severe psychological impact. Significant factors included gestational age, watching television for 2-4 hours per day, traveling to infected areas, and using social media for 1-2 hours per day. We recommend the following to concerned bodies, including the Somali Regional Health Bureau, health institutions, researchers, and the community:

- ✓ Thoughtful planning and timely preparation by the government are recommended to mitigate the negative impacts of the COVID-19 pandemic and restore the quality of life among pregnant women.
- ✓ Our findings suggest the development of psychological interventions and public mental health strategies integrated into pandemic response efforts early on to address the psychological needs of pregnant women.
- ✓ Further research is needed to investigate the long-term psychological effects of the COVID-19 pandemic on pregnant women, excluding those in the postpartum period.

## Data Availability

All data generated or analyzed during this study are included in this published article.

## Abbreviations

COVID-19: Corona Virus 2019
DASS-21: Depression Anxiety Stress Scale-21 Items
IES-R: Impact of Event Scale-Revised
OSS-3: Oslo-3 Social Support Scale
PTSD: Post Traumatic Stress Disorders
SARS-CoV-2: Severe Acute Respiratory Syndrome Coronavirus 2
